# Differential gene expression in tomato fruit and *Colletotrichum gloeosporioides* during colonization of the RNAi*–SlPH* tomato line with reduced fruit acidity and higher pH

**DOI:** 10.1186/s12864-017-3961-6

**Published:** 2017-08-04

**Authors:** Shiri Barad, Noa Sela, Amit K. Dubey, Dilip Kumar, Neta Luria, Dana Ment, Shahar Cohen, Arthur A. Schaffer, Dov Prusky

**Affiliations:** 1 Department of Postharvest Science of Fresh Produce, Agricultural Research Organization, the Volcani Center, 7505101 Rishon LeZion, Israel; 2 Department of Plant Pathology and Microbiology, The Robert H. Smith Faculty of Agriculture, Food and Environment, The Hebrew University of Jerusalem, 76100 Rehovot, Israel; 3 Department of Plant Pathology and Weed Research, ARO, the Volcani Center, 50250 Bet Dagan, Israel; 4 Department of Plant Sciences, Agricultural Research Organization, the Volcani Center, 50250 Bet Dagan, Israel

**Keywords:** Fungal pH regulation, Induced pathogenicity, Host pH regulation

## Abstract

**Background:**

The destructive phytopathogen *Colletotrichum gloeosporioides* causes anthracnose disease in fruit. During host colonization, it secretes ammonia, which modulates environmental pH and regulates gene expression, contributing to pathogenicity. However, the effect of host pH environment on pathogen colonization has never been evaluated. Development of an isogenic tomato line with reduced expression of the gene for acidity, *SlPH* (Solyc10g074790.1.1), enabled this analysis. Total RNA from *C. gloeosporioides* colonizing wild-type (WT) and RNAi–*SlPH* tomato lines was sequenced and gene-expression patterns were compared.

**Results:**

*C. gloeosporioides* inoculation of the RNAi–*SlPH* line with pH 5.96 compared to the WT line with pH 4.2 showed 30% higher colonization and reduced ammonia accumulation. Large-scale comparative transcriptome analysis of the colonized RNAi–*SlPH* and WT lines revealed their different mechanisms of colonization-pattern activation: whereas the WT tomato upregulated 13-LOX (lipoxygenase), jasmonic acid and glutamate biosynthesis pathways, it downregulated processes related to chlorogenic acid biosynthesis II, phenylpropanoid biosynthesis and hydroxycinnamic acid tyramine amide biosynthesis; the RNAi–*SlPH* line upregulated UDP-D-galacturonate biosynthesis I and free phenylpropanoid acid biosynthesis, but mainly downregulated pathways related to sugar metabolism, such as the glyoxylate cycle and L-arabinose degradation II. Comparison of *C. gloeosporioides* gene expression during colonization of the WT and RNAi–*SlPH* lines showed that the fungus upregulates ammonia and nitrogen transport and the gamma-aminobutyric acid metabolic process during colonization of the WT, while on the RNAi–*SlPH* tomato, it mainly upregulates the nitrate metabolic process.

**Conclusions:**

Modulation of tomato acidity and pH had significant phenotypic effects on *C. gloeosporioides* development. The fungus showed increased colonization on the neutral RNAi–*SlPH* fruit, and limited colonization on the WT acidic fruit. The change in environmental pH resulted in different defense responses for the two tomato lines. Interestingly, the WT line showed upregulation of jasmonate pathways and glutamate accumulation, supporting the reduced symptom development and increased ammonia accumulation, as the fungus might utilize glutamate to accumulate ammonia and increase environmental pH for better expression of pathogenicity factors. This was not found in the RNAi–*SlPH* line which downregulated sugar metabolism and upregulated the phenylpropanoid pathway, leading to host susceptibility.

**Electronic supplementary material:**

The online version of this article (doi:10.1186/s12864-017-3961-6) contains supplementary material, which is available to authorized users.

## Background

Acidity is a major determinant of fruit taste and quality, in combination with sugars and flavor volatiles. Most edible fruits have acidic pH values in the range of 3–5. Previous studies identified a gene family encoding membrane proteins responsible for acidity in fruit, termed *PH*, and showed functionality of the gene in tomatoes [1]. This suggested its importance for the fruit acidity trait. However, the mechanism underlying this gene’s modulation of pH is not fully understood. Cohen et al. [1] were the first to develop a stable RNA interference (RNAi) transgenic tomato line (RNAi–*SlPH*) with about 30% less citric acid and no significant differences in malic acid levels relative to the wild type (WT), resulting in tomatoes with more than a full unit higher pH [1].

The ability of postharvest pathogens to alter pH locally was initially described for *Colletotrichum gloeosporioides*, and then extended to some other pathogens, such as *Alternaria alternata*, *Botrytis cinerea*, *Penicillium expansum*, *Penicillium digitatum*, *Penicillium italicum, Phomopsis mangiferae, Monilinia fructicola*, and *Fusarium oxysporum* [2–10]. Attacking pathogenic fungi such as *P. expansum*, *P. digitatum*, *P. italicum* [7], *Phomopsis mangiferae* [2], *B. cinerea* [5], and *Sclerotinia sclerotiorum* [11] acidify tissue with organic acids. Fungi can also achieve ambient alkalization by actively secreting ammonia, which results from protease activation followed by amino acid deamination [12]. Ammonium accumulation has been detected in association with pathogenicity of many *Colletotrichum* species, including *C. gloeosporioides, C. acutatum, C. higgisianum, C. graminicola*, and *C. coccodes* [8, 13–15], as well as *A. alternata* [3, 4], and *F. oxysporum* [9]. The ammonium secreted by these species alkalizes the host tissue; its concentration can reach approximately 5 mM, as found in decayed avocado, tomato, and persimmon fruit [3, 4, 8, 13]. In each case with *Colletotrichum* spp., increased ammonium accumulation has been related to enhanced pathogenicity [8, 13, 16]. In the case of *A. alternata*, ammonium accumulation led to a 2.4 pH unit increase in several hosts—tomato, pepper, melon, and cherry [3, 4]. Interestingly, ammonia accumulation and pH increase were not correlated across host species, suggesting that pH increase in each host depends on a complex interaction that involves the buffering capacity of the tissue, nitrogen and carbon availability, and the fruit’s initial pH [4]. However, low pH has been found to activate higher ammonia production and secretion in *Colletotrichum* spp. [13, 17].

In both cases, i.e., alkalization and acidification of the environment via secretion of ammonia by *Colletotrichum* and organic acid by *Penicillium*, respectively, pathogenicity factors are clearly modulated, being either activated or repressed [18, 19]. *P. expansum* acidifies the host tissue to pH levels of 3.5 to 4.0, conditions that significantly enhance polygalacturonase (*pg1*) transcription [7, 19]. Similarly, in *C. gloeosporioides*, *pelB*, encoding pectate lyase, is expressed and secreted in vitro at pH levels higher than 5.7, similar to the pH values present in decaying tissue [20–22]. This suggests that postharvest pathogens modulate the expression of genes contributing to pathogenicity according to environmental pH-inducing conditions.

The development of the RNAi–*SlPH* tomato line, which has reduced acidity and increased pH relative to the WT [1, 23], offered the possibility of testing the effect of host changes on the pathogenicity factors affecting *C. gloeosporioides* colonization [24]. The published transcriptomic analyses of the tomato fruit and the pathogen responses during fungal colonization are the basis for the present analysis [24, 25]. Previously, we analyzed the importance of the small molecules secreted by the pathogen as key factors modulating environmental pH and activating fungal colonization of a single host. In the present work, transcriptomic analysis of two similar hosts—WT and a transgenic tomato line with downregulation of a single gene affecting acidity and pH—showed that pH affects not only the pathogen but also the host’s gene expression and the phenotypic host response to fungal colonization.

## Results and discussion

### Analysis of pH, acidity and susceptibility of RNAi–*SlPH* fruit colonized by *C. gloeosporioides*

Comparison of freshly harvested tomato fruits of the RNAi–*SlPH* and WT lines showed that the former has threefold less total acid than the latter (Table 1). Evaluation of the mesocarp tissue of RNAi–*SlPH* fruit showed significantly higher pH values than in the WT containing the *SlPH* gene (Table 1).

**Table 1:** pH and total acids (TA) of healthy WT and RNAi–*SlPH* tomato lines

	WT	RNAi–*SlPH*
pH	4.17 ± 0.04	5.96 ± 0.02
(%)TA	0.85 ± 0.04	0.29 ± 0.02

**Fig. 1:**
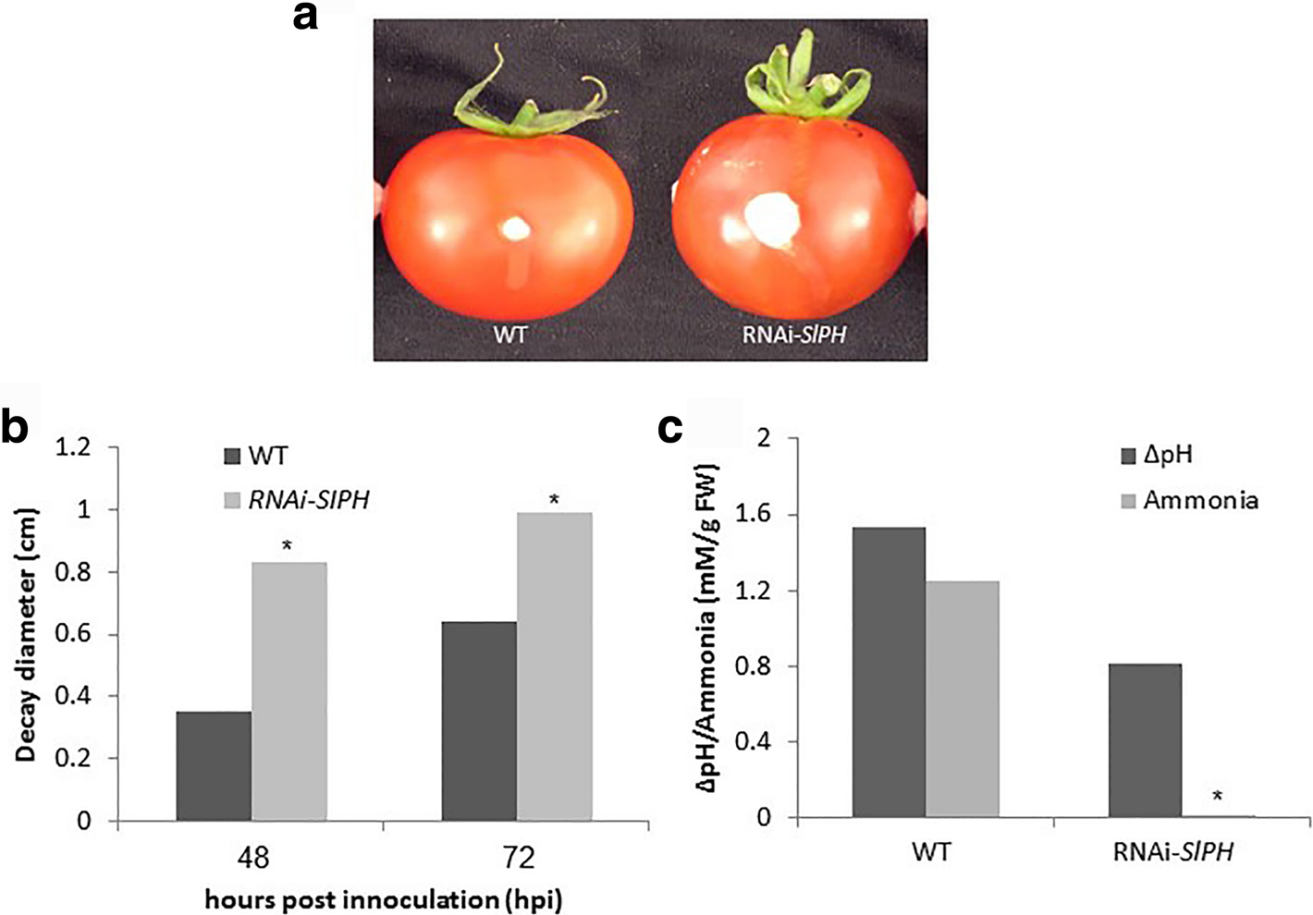
Colonization patterns, ammonia accumulation and pH changes induced by *C. gloeosporioides* on WT and RNAi–*SlPH* tomato lines. **a** WT and RNAi–*SlPH* tomato tissue colonized by *C. gloeosporioides* 3 days postinfection (PI). **b** Decay development on the two infected tomato *lines* (average of four to five infected fruits reported). **c** Ammonia accumulation and pH changes in the WT compared to the RNAi–*SlPH* tomato *lines*. For inoculation, 10 μl of 10^6^ spore ml^−1^ suspension was placed in 1-mm deep, 2-mm diameter holes, and incubated at 25 °C under high humidity. Average ± SD of four to five replicates of one experiment out of three repeated experiments is presented

Comparison of *C. gloeosporioides* isolate Cg-14 colonization patterns on freshly harvested WT and RNAi–*SlPH* fruit inoculated with a suspension of 10^6^ spore ml^−1^ showed 2.37- and 1.54-fold enhanced colonization of the RNAi–*SlPH* fruit after 48 and 72 h, respectively, compared to the WT. Ammonia accumulation in the mesocarp of the RNAi–*SlPH* line was significantly lower than that detected in the WT mesocarp (0.002 compared to 1.255 mM). Ammonia accumulation was accompanied by an increase in pH (∆pH of 1.5 in the WT line compared to ∆pH 0.8 in the RNAi–*SlPH* line), suggesting that fungal colonization of the RNAi–*SlPH* line occurs with reduced ammonia accumulation (Fig. 1) as a result of an enhanced pathogen and/or host response induced by that strain’s higher pH level which contributes to fungal colonization.

### Profiling gene expression in RNAi–*SlPH* and WT tomato fruit during colonization by *C. gloeosporioides*

Gene profiling was carried out to determine the factors in the host and pathogen response that modulate the two tomato lines differential behavior. Gene-expression profiling of tomato fruit was conducted in two replicates of non-inoculated and *C. gloeosporioides*-inoculated WT and RNAi–*SlPH* lines. To determine the effect of initial host pH on fungal responses, eight libraries of single-end RNA sequences (deposited in the NCBI Sequence Read Archive (SRA)) under accession no. SRP078571) were mapped to the reference genomes of tomato (*S. lycopersicum*, build 2.50) [23] and *C. gloeosporioides* isolate Cg-14 (GEO accession number GSE41844) [24] using Bowtie2 software [26]. The eight libraries consisted of: (i) two replicates of a healthy WT tomato line; (ii) two replicates of a WT tomato line infected with *C. gloeosporioides* sampled 72 h postinoculation; (iii) two replicates of a healthy RNAi*–SlPH* tomato line, and (iv) two replicates of an RNAi*–SlPH* tomato line infected with *C. gloeosporioides* 72 h postinoculation.

**Fig. 2:**
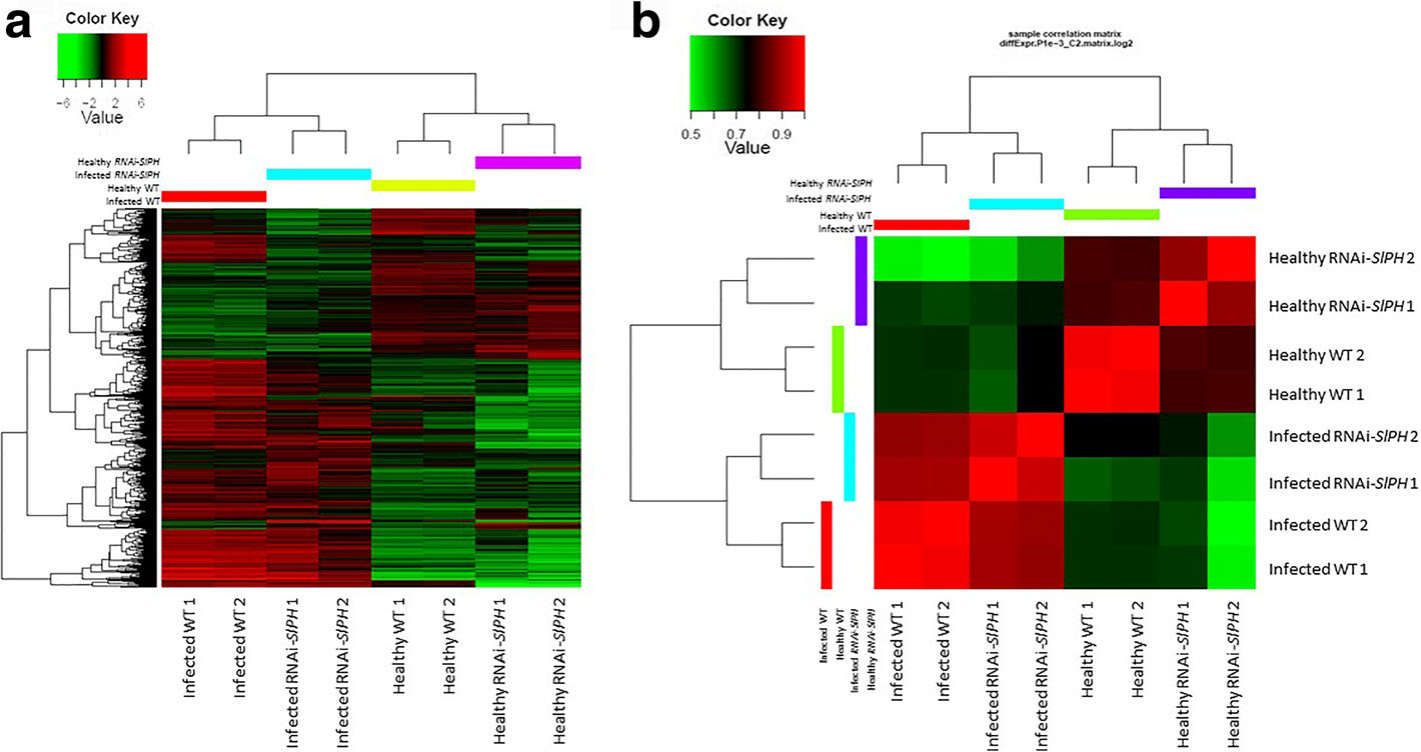
**a** Heat map of Pearson correlation between expression levels of genes in different samples. *Red* – highly correlated samples, *green* – low correlation. The correlation was calculated using the R ‘cor’ function and visualized using the ‘ggplots’ package. **b** Heat map of expression matrix logCPM value (which is log2 counts per million, normalized for library sizes) clustered for both genes and samples

**Fig. 3:**
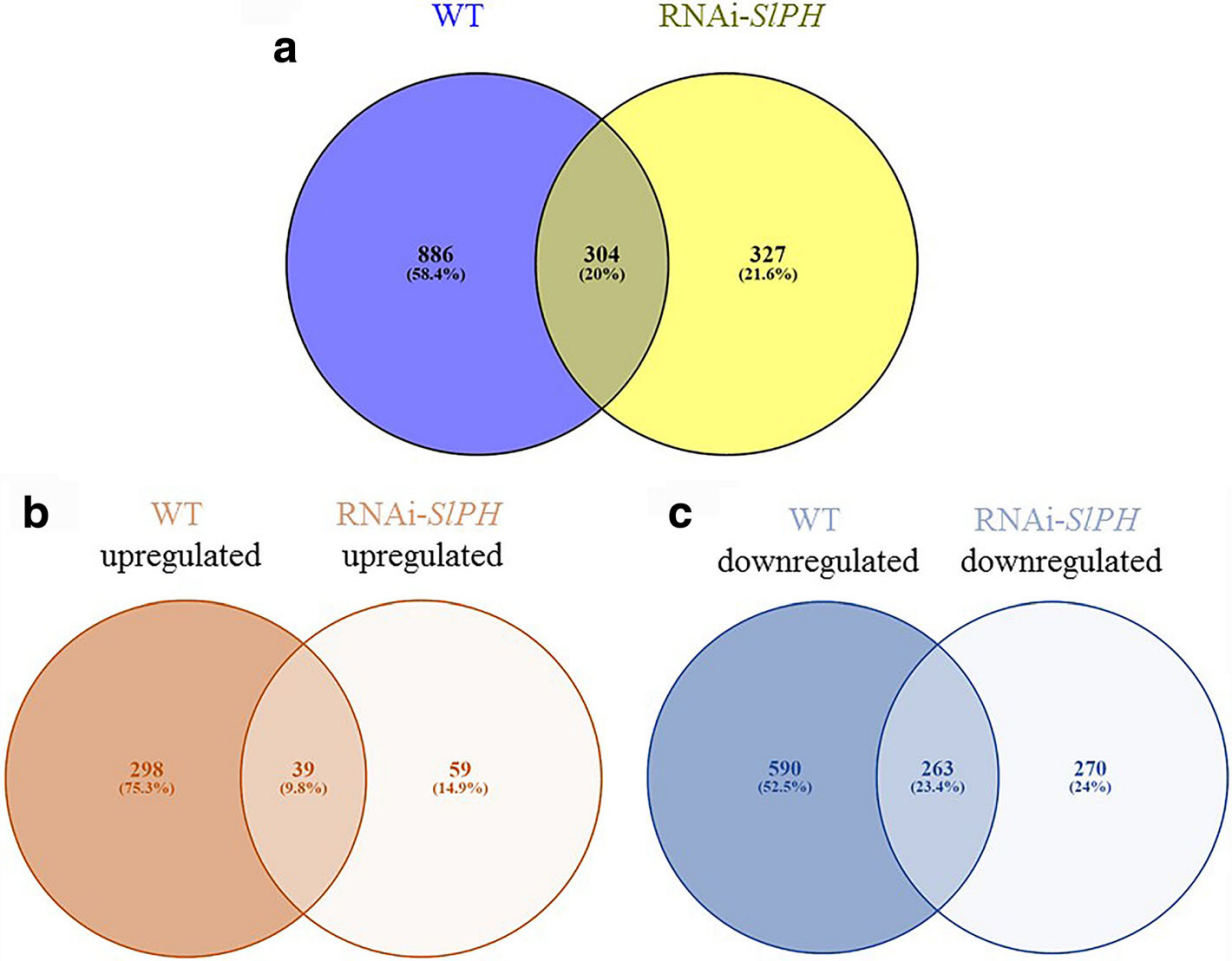
**a** Venn diagram of differential gene expression between healthy vs. *C. gloeosporioides*-infected WT tomato *line* and healthy vs. *C. gloeosporioides*-infected RNAi–*SlPH* tomato *line*. **b** Upregulated expression. **c** Downregulated expression. The Venn diagrams were calculated using VENNY 2.1 software [72]

Hierarchical clustering analysis of the tomato lines indicated different gene-expression patterns in healthy vs. infected tissues. Overall, there were more upregulated than downregulated genes upon *C. gloeosporioides* infection in both lines (Fig. 2a). By edgeR analysis [27], 1190 and 631 genes were differentially expressed in the healthy vs. infected WT and healthy vs. infected RNAi–*SlPH* lines, respectively (Fig. 3), with a false discovery rate (FDR) threshold of <1e^−3^ and Log “Fold Change” (FC) greater than 2 or smaller than −2. Thus the WT line showed an almost sixfold increase in gene expression compared to the RNAi*–SlPH* line, suggesting negative regulation of gene expression in the RNAi*–SlPH* line.

Pearson correlation analysis between the expression levels of genes in the different samples showed a high degree of agreement between measurements conducted on replicates of each treatment, indicating the reproducibility of the results (see experimental correlation heat map in Fig. 2b). Gene-expression levels for healthy WT and RNAi*–SlPH* tomatoes clustered together, as did the levels for infected WT and RNAi*–SlPH* tomatoes. The heat map further showed that the major differences in gene expression were between infected and non-infected tomato lines, whereas only minor differences were due to introduction of the mutation in the RNAi*–SlPH* line.

### Analysis of the differentially expressed tomato genes during *C. gloeosporioides* infection

To understand the importance of the specific changes in both cultivars, the differentially expressed genes in both lines were analyzed. Of the 1190 and 631 genes that were differentially expressed during fungal colonization of WT and RNAi*–SlPH* tomato lines, respectively, 304 (20%) were similar between lines (Fig. 3a); 886 genes were exclusively differentially expressed during infection of the WT line and may contribute to this line’s reduced susceptibility compared to the RNAi*–SlPH* line (Fig. 3a). Of these 888 genes, 298 were upregulated and 590 were downregulated (Fig. 3b and c). However, only 329 genes were exclusively differentially expressed during infection of RNAi*–SlPH* tomato, possibly contributing to this line’s susceptibility (Fig. 3a). Of these, 59 were upregulated and 270 were downregulated (Fig. 3b and c). The difference in gene profiles between the high and low pH lines might indicate that different processes are modulated by the same pathogen under initial conditions of differential host pH.

These results suggested that some of the 298 upregulated and 590 downregulated genes in the colonized WT line might contribute to reduced fungal development (Fig. 3b). Similarly, some of the 59 upregulated and 270 downregulated genes might contribute to the increased susceptibility to colonization of the RNAi–*SlPH* line (Fig. 3c). Furthermore, the host response might be strongly affected by the initial pH. If we consider that tomato fruit pH can increase from harvesting at the breaker stage (initial ripening) to full maturity, pH may strongly affect host responses to pathogen on the same fruit.

### Differentially regulated host pathways during infection of the WT and RNAi–*SlPH* lines

Using MetGenMap software [28], we discovered the changed pathways (KEGG) in each tomato line during infection.

#### Pathways modulating host resistance: Jasmonic acid (JA) and phenylpropanoid

The most strongly upregulated pathway in the colonized WT line was the 13-LOX and 13-HPL (hydroperoxide lyase pathway) pathway involved in 13-LOX activity (Table 2). These enzymes are involved in the biosynthesis of JA during the transformation of linolenic acid to JA by a multistep process [29]. JA has been proven to be involved in plant resistance to pathogens by activating pathogenesis-related proteins such as PR-1, PR-3 and PR-8 [30]. This pathway was not significantly upregulated during infection of the RNAi*–SlPH* line, suggesting that this upregulation may contribute to the reduced colonization of the WT line compared to the RNAi–*SlPH* line.

**Table 2:** Upregulated and downregulated pathways in *C. gloeosporioides*-infected WT lines compared to RNAi–*SlPH* lines

	Pathway name	*P*-value
Upregulated	13-LOX and 13-HPL pathway	0.0074119
	oleate biosynthesis I (plants)	0.0113267
	jasmonic acid biosynthesis	0.0144936
	wax ester biosynthesis	0.0170085
	glutamate biosynthesis IV	0.0253189
Downregulated	phenylethanol biosynthesis	2.302E-05
	ethylene biosynthesis from methionine	0.0002738
	suberin biosynthesis	0.0005621
	simple coumarin biosynthesis	0.0019152
	hydroxycinnamic acid tyramine amide biosynthesis	0.0022188
	chlorogenic acid biosynthesis II	0.0027091
	wound-induced proteolysis I	0.0086595
	alanine biosynthesis III	0.0086595
	seed germination protein turnover	0.0086595
	purine degradation	0.0094942
	phenylpropanoid biosynthesis, initial reactions	0.0136983
	fatty acid α-oxidation	0.0160793
	aesculetin biosynthesis	0.0174724
	glutamate degradation II	0.0422702
	ternatin C5 biosynthesis	0.0422702

In contrast, a pathway that was downregulated during *C. gloeosporioides* infection of the WT line was the chlorogenic acid biosynthesis II pathway (Table 2). Chlorogenic acid (5-O-caffeoyl-D-quinic acid) is one of the most widespread hydroxycinnamic acid derivatives in plants. Its physiological roles include defense, disease resistance as an antioxidant, and growth regulation [31]. Moreover, hydroxycinnamic acid tyramine amide biosynthesis was also downregulated in the infected WT tomato line (Table 2). Incorporation of hydroxycinnamic acid tyramine amides into the cell wall has been reported to enhance its efficiency as a barrier against pathogens by increasing its rigidity and decreasing its digestibility [32–34]. This type of response has been observed during colonization of potato by *Phythopthora infestans*, with the potato tyramine N- hydroxycinnamoyl transferase activity increasing upon infection, as well as upon wounding [34], and during appressorium formation at the biotrophic stage of *C. gloeosporioides* [25], although it may not affect the response of the WT strain. The chlorogenic acid biosynthesis II pathway was not modulated by *C. gloeosporioides* in the RNAi–*SlPH* line*.* It is possible that under the new environmental conditions present in this line, the fungus does not modulate this defense system and/or has a different, more efficient mechanism to enable its enhanced colonization.

#### Pathways modulating nitrogen metabolism: Glutamate and glutamine metabolism

An important pathway that was upregulated during colonization of the WT line by *C. gloeosporioides* was the glutamate biosynthesis IV pathway, along with downregulation of the glutamate degradation II pathway (Table 2). The glutamine synthetase–glutamine-oxoglutarate aminotransferase (GS–GOGAT) cycle has been proposed to function in primary nitrogen assimilation, in which glutamate is continuously synthesized by the GOGAT glutamate synthase and metabolized by the enzyme GS [35–38]. Glutamate metabolism has a pivotal role in plant amino acid metabolism since it orchestrates crucial metabolic functions, including assimilation or dissimilation of ammonia and amino acid transamination, and it provides both the carbon skeleton and α-amino group for biosynthesis of amino acids with key roles in plant defense, such as γ-aminobutyric acid (GABA), arginine, and proline [36, 39]. It is therefore responsible for cell viability.

The host glutamate metabolism in the WT strain may function in two opposing ways in response to pathogens, either activating the host defense response, or being exploited by the pathogen to promote infection, for example by ammonia accumulation [8, 40]. It has been proposed that nitrogen accumulation in the colonized area leads to strong resistance to the pathogen. Overexpression of glutamate receptors in transgenic *Arabidopsis* plants increased ammonium transportation within the challenged cells, resulting in delayed senescence and increased levels of resistance against *B. cinerea* [41]. This may also explain the observed levels of resistance in *Arabidopsis* lines overexpressing arginase, a urea-generating enzyme which eventually supplies the GS–GOGAT cycle with nitrogen sources [40, 42]. This suggests that the high level of GS activity maintains the critical functionality of the GS–GOGAT cycle at the inoculated sites. Similarly, upregulation of GS1 and accumulation of glutamine at an early infection stage demonstrated that nitrogen remobilization is stimulated in infected leaves [43]. Collectively, it seems that glutamate might promote activation of the GS–GOGAT cycle to boost tolerance to the pathogen in infected tissues.

Modulation of the glutamate biosynthesis pathway during *C. gloeosporioides* infection of a WT line has also been reported in the interaction between *Colletotrichum lindemuthianum* and *Phaseolus vulgaris*. In that system, upon pathogen colonization, GS1 levels increase in the host during the biotrophic stage of *Colletotrichum*, resulting in glutamine accumulation in the phloem around the infection site. Concomitantly, *C. lindemuthianum* shifts to necrotrophic invasion, presumably because the increased vascular glutamine concentration is perceived as a host escape signal [40, 43]. This might explain the relatively limited colonization pattern observed here on the WT tomato compared to the RNAi–*SlPH* line. This timely transition in virulence strategy enables the anthracnose pathogen to trap high levels of glutamine in the phloem before the host can efficiently translocate its nitrogen reservoir out of the infected area.

These results indicate that during the coevolution of pathogenic fungi with plants, they adapted to the modifications in plant nitrogen content caused by biotic stress, ultimately turning the metabolic changes in the plant to their benefit [44, 45]. This suggests that *C. gloeosporioides* exploits nitrogen metabolism differently in the two tomato lines. During infection of the WT and its enhanced glutamate synthesis, the fungus upregulates genes involved in glutamate metabolism to α-ketoglutarate (fungal *gdh2*) (Table 3, Fig. 6). This might result in continuous glutamate degradation by the fungus during infection and ammonia accumulation; at the same time, the host uses this process to escape attack. In the case of the infected RNAi–*SlPH* line, there is no upregulation of glutamate biosynthesis, thereby blocking the host defense system, and enabling fungal development without ammonia accumulation. This suggests that ammonia accumulation via fungal degradation of glutamate determines not only the necrotrophic stage but also the host response to the dynamics of glutamate transformation and the preservation of cell viability.

**Table 3:** Upregulated and downregulated pathways in *C. gloeosporioides*-infected RNAi–*SlPH* lines compared to WT lines

	Pathway name	*P*-value
Upregulated	matairesinol biosynthesis	0.0049779
	UDP-D-galacturonate biosynthesis I (from UDP-D-glucuronate)	0.0165126
	free phenylpropanoid acid biosynthesis	0.0246834
	gibberellin inactivation	0.0408552
Downregulated	glyoxylate cycle	0.0012551
	glycolate and glyoxylate degradation II	0.0015753
	cytokinin degradation	0.003706
	superpathway of glyoxylate cycle	0.0070379
	wax ester biosynthesis II	0.0117932
	anthocyanin biosynthesis (delphinidin 3-O-glucoside)	0.0181857
	L-arabinose degradation II	0.0209389
	anthocyanin biosynthesis (pelargonidin 3-O-glucoside, cyanidin 3-O-glucoside)	0.0218204
	leucopelargonidin and leucocyanidin biosynthesis	0.0263502
	leucodelphinidin biosynthesis	0.0263502
	glycogen degradation I	0.0320831
	melibiose degradation	0.0414508

#### Pathways modulating sugar metabolism

The RNAi–*SlPH* tomato showed upregulation and downregulation of sugar metabolic pathway genes during colonization by *C. gloeosporioides*: differential upregulation of the UDP-galacturonate biosynthesis pathway as well as downregulation of the glyoxylate cycle, glyoxylate degradation, superpathway of glyoxylate cycle, glycogen degradation, and arabinose degradation, suggesting a very specific decrease in sugar metabolism (Table 3). The glyoxylate cycle converts acetyl-CoA to succinate for the synthesis of carbohydrates [46]; organisms with a glyoxylate cycle therefore gain metabolic versatility because it allows cells to utilize simple carbon compounds as a carbon source when complex sources such as glucose are not available. Glyoxylate derivatives can accumulate in plants under stress; they react with DNA, oxidize membrane lipids, modify proteins or influence the transcription of stress-related genes, thereby causing cellular and developmental problems that lead to host susceptibility [47–49]. The drop in glyoxylate pathway activity in RNAi–*SlPH* fruit may result in decreased levels of malic and citric acid. This reduction in organic acids may lead to enhanced neutralization of the tissue environment, and upregulation of the *C. gloeosporioides* transcription factor regulating pH-affected genes *pacC* and *pelB*, which are expressed at high pH levels without ammonia accumulation. This negative regulation of pH environment (i.e., not by increasing ammonia but by reducing organic acid accumulation) might explain the increased colonization of *C. gloeosporioides* in the RNAi–*SlPH* strain in the presence of reduced ammonia accumulation. This possibility is supported by the recent suggestion by Bi and coworkers [50] that *C. gloeosporioides* produces both ammonia and gluconic acid under different host/sugar conditions.

### Inversely regulated pathways during infection of the WT and RNAi–*SlPH* lines: Phenylpropanoid pathway

The phenylpropanoids are not only indicators of plant stress responses upon variations in light or mineral treatment; they are also key mediators of plant resistance as they are formed during the initial response to infection [32, 51, 52]. In our case, *C. gloeosporioides* repressed the phenylpropanoid pathway in the WT line, but upregulated it in the RNAi*–SlPH* line (Tables 2 and 3). Phenylalanine ammonia-lyase (PAL) is the first enzyme of the phenylpropanoid pathway, which synthesizes trans-cinnamic acid, a precursor of salicylic acid (SA) [53]. SA is a plant signal for the activation of defense responses and enhances host cell death [54]. During the interaction of soybean and the pathogen *Pseudomonas syringae* pv. *glycinea,* addition of physiological concentrations of SA enhanced the induction of defense gene transcripts, H_2_O_2_ accumulation, and hypersensitive cell death by an avirulent strain of the pathogen [55]. In our case, the necrotrophic stage of *C. gloeosporioides* may thrive under cell-death conditions. This is probably the contribution of the phenylpropanoid pathway to the enhanced pathogenicity in the RNAi–*SlPH* line, whereby overexpression of those genes that contribute to host cell death shifts the balance from resistance to susceptibility, as observed in this specific line. Future analysis of cell viability should fully confirm the present suggestions.

### Differentially regulated fungal genes during infection of the WT and RNAi*–SlPH* lines

**Fig. 4:**
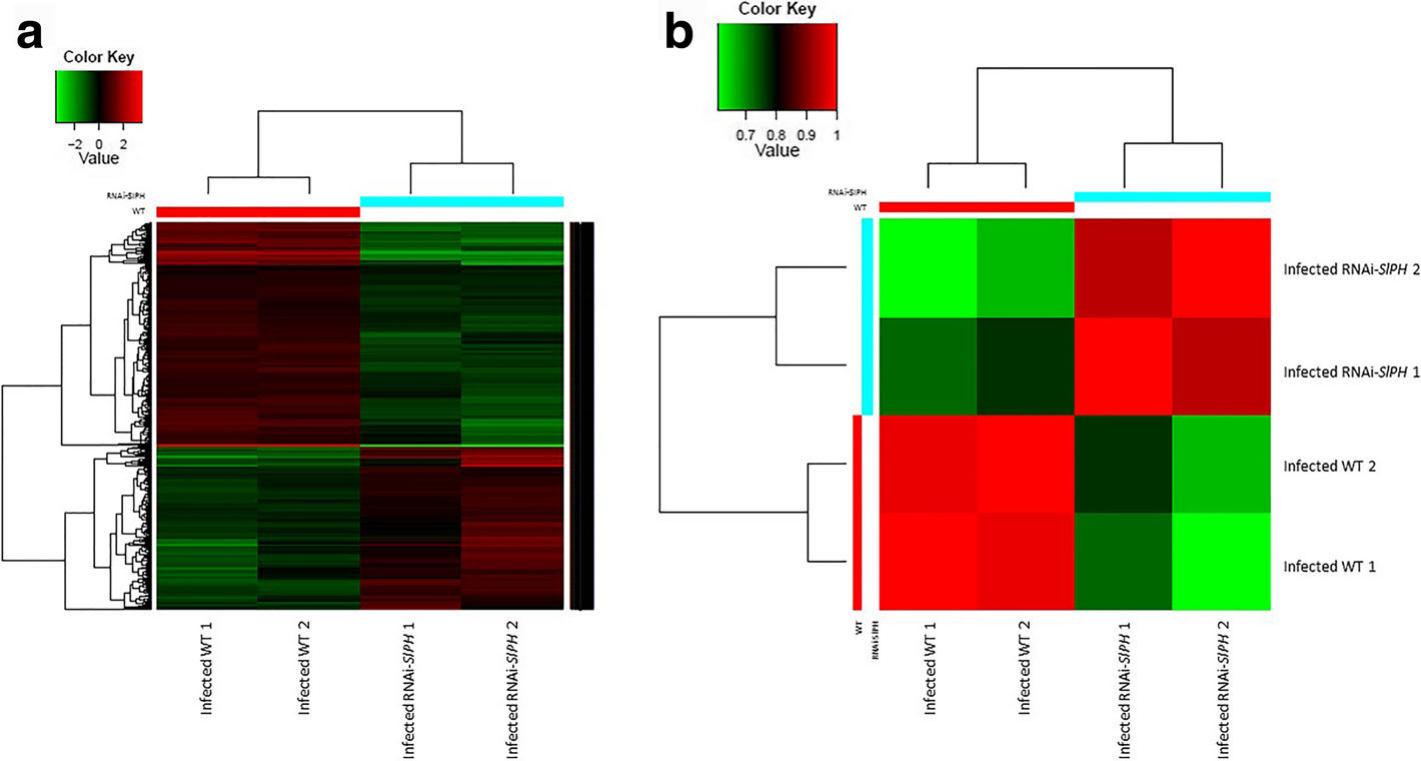
**a** Heat map of Pearson correlation analysis between all differentially expressed fungal genes of libraries from *C. gloeosporioides*-infected WT and RNAi–*SlPH* *lines* with threshold described in the manuscript. **b** Heat map of differentially expressed genes

Hierarchical clustering analysis of fungal gene expression during pathogenicity on WT and RNAi*–SlPH* tomatoes indicated different expression patterns on each infected line. Overall, there were more upregulated than downregulated *C. gloeosporioides* genes during infection of the WT vs. RNAi*–SlPH* line (Fig. 4a). Using edgeR analysis [27], 645 genes were differentially expressed in *C. gloeosporioides* during infection of the WT compared to infection of the RNAi–*SlPH* line, with a FDR threshold <0.05 and FC greater than 2 or smaller than −2. Among the differentially expressed genes, 415 were downregulated during infection of the RNAi*–SlPH* line and 230 were upregulated (Fig. 4).

Pearson correlation analysis of the expression levels of *C. gloeosporioides* transcripts in the different samples can serve for quality control of experimental reproducibility. The analysis showed strong agreement between measurements conducted on replicates of the various treatments, indicating high reproducibility of the results (see experimental correlation heat map in Fig. 4b).

### Modulation of fungal gene ontology (GO) categories during infection of RNAi–*SlPH* tomato lines

The key GO response in *C. gloeosporioides* during infection of the RNAi*–SlPH* lines was upregulation of fungal nitrate metabolic processes. These processes are activated when the preferred products—ammonia, glutamine, asparagine or glutamate—are not available. *In planta*, nitrogen availability seems to be limiting [56], indicating the fungus’ need for nitrate metabolism (Tables 4 and 5). Utilization of nitrate requires de novo synthesis of nitrate and nitrite reductase, which requires both nitrogen derepression and specific induction by nitrate. Fungal *nit* regulation is important for pathogenicity in *Colletotrichum* because it contributes to ammonia accumulation [13]. However, in our case, ammonia did not accumulate with the RNAi–*SlPH* line. Another possible explanation for the lack of fungal ammonia secretion with the RNAi*–SlPH* line is downregulation of ammonia transporters that are not active at the host’s higher pH. While ammonia accumulation was upregulated during infection of the WT, it was downregulated during infection of the RNAi*–SlPH* line (Fig. 1). This may result from inhibition of fungal export of ammonia and possible accumulation of ammonia inside the hyphae, as occurs in the spores of *∆mep* (ammonia permease) strains [57]. We did not monitor ammonia accumulation in *C. gloeosporioides* developing on the RNAi*–SlPH* tomato; however, downregulation of *mepB* was observed in this line (Table 3), suggesting that possibility. Furthermore, strong inhibition of NAD^+^-specific glutamate dehydrogenase (GDH2, encoding ammonia synthesis, JN660152) and ammonia transporter (AMET), which are downregulated at pH 6.0–7.0, supports the behavior in the RNAi*–SlPH* line [8, 58]. Thus, the differential activation of nitrogen metabolism by the pathogen is probably modulated differently in the WT and RNA–*SlPH* lines. These results suggested that the pathogen enhances colonization at a higher pH in the (RNAi–*SlPH* line without ammonia accumulation as a result of the decrease in *mepB* expression. Together, the lack of ammonia accumulation, the concurrent drop in glyoxylate pathway activity and the possible reduction of organic acid accumulation may contribute to fungal colonization by *C. gloeosporioides*.

**Table 4:** Upregulated fungal GO categories during *C. gloeosporioides* infection of RNAi–*SlPH* lines

GO term	Gene description
nitrate metabolic process	nitrate reductase
	nitrite reductase
	nia_beaba ame: full = nitrate reductase short = nr

**Table 5:** Upregulated fungal GO categories during *C. gloeosporioides* infection of the WT lines

GO term	Gene description
nitrogen compound transport	choline transport
	amino acid permease
	purine-cytosine permease
	choline transport
	---NA---
	choline transport
	ncs1 nucleoside transporter
	urea active transporter
	amino-acid permease inda1
	amino acid permease
	c6 finger domain
	amino-acid permease inda1
	amino acid
	urea transporter
	hypothetical protein [Tuber melanosporum Mel28]
	cytosine-purine permease
	amino acid
	amino acid permease
	mfs peptide putative
	peptide transporter ptr2-a
	ammonium transporter mep1
	dicarboxylic amino acid permease
	amino acid permease
	ammonium transporter
	large neutral amino acids transporter small subunit 1
	ammonium transporter mep2
	uridine permease
	amino-acid permease inda1
	proline-specific permease
	amino acid permease family
	amino acid permease
	uracil permease
	peptide transporter ptr2-a
	ncs1 nucleoside transporter family protein
	choline transport protein
	polyamine transporter tpo5
	ammonium transporter
	amino acid permease
	uracil permease
	ncs1 allantoate transporter
	ncs1 allantoate transporter
	tpa: amino acid transporter
anion transmembrane transport	choline transport
	amino acid permease
	choline transport
	---NA---
	choline transport
	amino-acid permease inda1
	amino acid permease
	c6 finger domain
	amino-acid permease inda1
	amino acid
	amino-acid permease inda1
	proline-specific permease
	amino acid permease family
	amino acid permease
	amino acid
	amino acid permease
	choline transport protein
	polyamine transporter tpo5
	amino acid permease
	dicarboxylic amino acid permease
	amino acid permease
	mitochondrial phosphate carrier protein
	tpa: amino acid transporter
	large neutral amino acids transporter small subunit 1
ion transmembrane transport	choline transport
	amino acid permease
	choline transport
	---NA---
	choline transport
	membrane zinc transporter
	amino-acid permease inda1
	amino acid permease
	potassium transporter
	c6 finger domain
	amino-acid permease inda1
	amino acid
	p-type calcium ATPase
	amino acid
	amino acid permease
	ammonium transporter mep1
	dicarboxylic amino acid permease
	amino acid permease
	ammonium transporter
	---NA---
	large neutral amino acids transporter small subunit 1
	ammonium transporter mep2
	potassium uptake transporter
	amino-acid permease inda1
	proline-specific permease
	amino acid permease family
	amino acid permease
	choline transport protein
	polyamine transporter tpo5
	ammonium transporter
	amino acid permease
	mitochondrial phosphate carrier protein
	tpa: amino acid transporter
oxidation-reduction process	general amidase
	cytochrome p450
	glyoxylate reductase
	short chain
	short-chain
	flavin-containing amine
	cytochrome p450
	FAD dependent
	D-amino acid
	2-deoxy-D-gluconate 3-dehydrogenase
	aryl-alcohol dehydrogenase
	3-ketoacyl-acyl carrier protein
	polyketide synthase
	glycerate dehydrogenase
	aldehyde dehydrogenase
	D-amino-acid oxidase
	sorbitol dehydrogenase
	FAD binding domain-containing protein
	trichothecene c-15 hydroxylase
	trichothecene c-15 hydroxylase
	short-chain dehydrogenase reductase
	copper amine oxidase
	xanthine dehydrogenase
	integral membrane protein
	cytochrome p450 monooxygenase
	integral membrane protein
	superoxide dismutase
	chlorocatechol -dioxygenase
	NADPH dehydrogenase
	hypothetical protein [Podospora anserina S mat+]
	2og-fe oxygenase superfamily protein
	cysteine dioxygenase
	cytochrome b5
	isocitrate dehydrogenase
	terpene synthase metal binding domain protein
	benzoate 4-monooxygenase cytochrome p450
	copper amine oxidase
	naphthalene -dioxygenase subunit alpha
	succinate-semialdehyde dehydrogenase
	Fe-containing alcohol
	copper amine oxidase
	short chain dehydrogenase
	cytochrome p450
	alcohol dehydrogenase
	FAD binding domain protein
	aldehyde dehydrogenase family
	l-ornithine 5-monooxygenase
	short-chain dehydrogenase reductase family
	proline oxidase
	potassium uptake transporter
	peroxisomal copper amine oxidase
	benzoate 4-monooxygenase cytochrome p450
	pigment biosynthesis protein ayg1
	FAD dependent oxidoreductase
	histidine acid phosphatase
	alpha-ketoglutarate dependent xanthine dioxygenase
	NAD-specific glutamate dehydrogenase
	amino acid transporter
	NAD-specific glutamate dehydrogenase
	bifunctional purine biosynthesis protein
	FAD dependent oxidoreductase superfamily
	short-chain dehydrogenase reductase sdr
	alcohol dehydrogenase
	glycine dehydrogenase
	aldehyde dehydrogenase
	short-chain dehydrogenase
	l-amino-acid oxidase
	NADP-specific glutamate dehydrogenase
	aerobactin siderophore biosynthesis protein iucb
	endoribonuclease l-psp
	cytochrome p450 oxidoreductase
	ferric-chelate reductase
	cytochrome b2
	aerobactin siderophore biosynthesis protein iucb
	short chain dehydrogenase
	glyoxylate reductase
	short-chain dehydrogenase reductase sdr
	peroxisomal dehydratase
	FAD binding domain-containing protein
	FAD binding domain-containing protein
	gtp-binding protein
	glycerate-and formate-dehydrogenase
	copper amine oxidase
	cytochrome b2
	cytochrome b2
	salicylate hydroxylase
	multicopper oxidase
	3-oxoacyl-(acyl-carrier-protein) reductase
	transcriptional activator protein acu-
	membrane copper amine
	---NA---
	dimethylglycine oxidase
	sarcosine oxidase
	FAD dependent oxidoreductase superfamily
	succinate-semialdehyde dehydrogenase
	aldehyde dehydrogenase
	rieske 2fe-2 s family protein
	short chain dehydrogenase
	rieske 2fe-2 s family protein
	ankyrin repeat-containing protein
	short chain dehydrogenase
	saccharopine dehydrogenase
	2og-fe oxygenase
	FAD binding domain containing protein
	major facilitator superfamily transporter
	hypothetical protein GLRG_11952 [Glomerella graminicola M1.001]
	aminobenzoyl-glutamate utilization protein b
	12-oxophytodienoate reductase
	---NA---
	Mfs
	FAD binding domain-containing protein
	benzoate 4-monooxygenase cytochrome p450
	FAD binding domain protein
	FAD dependent oxidoreductase superfamily
	cytochrome p450 alkane
	2og-fe oxygenase family
	oxidoreductase domain containing protein
	c6 transcription
	2og-fe oxygenase family
	short-chain dehydrogenase reductase
	3-hydroxyacyl-NAD binding
	alcohol dehydrogenase
	FAD binding domain-containing protein
	FAD binding domain-containing protein
	alcohol dehydrogenase
	Aldehyde
	cytochrome p450 alkane
	short chain dehydrogenase
	n-alkane-inducible cytochrome p450
	streptomycin biosynthesis protein
	methylmalonate-semialdehyde dehydrogenase
	flavin dependent
	Carbonyl
	2og-fe oxygenase superfamily protein
	short chain dehydrogenase reductase family
	homoisocitrate dehydrogenase
	flavin-binding monooxygenase
	phosphoadenosine phosphosulfate reductase
	phosphoadenosine phosphosulfate reductase
	sarcosine oxidase
	D-lactate mitochondrial precursor
	FAD-binding domain protein
	alpha-ketoglutarate dependent xanthine dioxygenase
ammonium transmembrane transport	ammonium transporter
	ammonium transporter mep2
	ammonium transporter mep1
	ammonium transporter
gamma-aminobutyric acid metabolic process	succinate-semialdehyde dehydrogenase
	4-aminobutyrate aminotransferase
	4-aminobutyrate aminotransferase
	4-aminobutyrate aminotransferase
	succinate-semialdehyde dehydrogenase
ion transport	choline transport
	amino acid permease
	choline transport
	---NA---
	choline transport
	membrane zinc transporter
	urea active transporter
	amino-acid permease inda1
	amino acid permease
	potassium transporter
	c6 finger domain
	amino-acid permease inda1
	amino acid
	hypothetical protein [Tuber melanosporum Mel28]
	p-type calcium ATPase
	plasma membrane h + −ATPase pma1
	amino acid
	amino acid permease
	ammonium transporter mep1
	dicarboxylic amino acid permease
	amino acid permease
	zip zinc transporter
	udp-galactose transporter
	ammonium transporter
	---NA---
	large neutral amino acids transporter small subunit 1
	ammonium transporter mep2
	potassium uptake transporter
	amino-acid permease inda1
	proline-specific permease
	amino acid permease family
	amino acid permease
	choline transport protein
	polyamine transporter tpo5
	ammonium transporter
	amino acid permease
	mitochondrial phosphate carrier protein
	tpa: amino acid transporter
organonitrogen compound catabolic process	urea active transporter
	proline oxidase
	---NA---
	methylmalonate-semialdehyde dehydrogenase
	urease
	glycosyl family
	succinate-semialdehyde dehydrogenase
	dimethylglycine oxidase
	NAD-specific glutamate dehydrogenase
	succinate-semialdehyde dehydrogenase
	dihydropyrimidinase
	4-aminobutyrate aminotransferase
	NAD-specific glutamate dehydrogenase
	aldehyde dehydrogenase
	3-hydroxyacyl-NAD binding
	carbamoyl-phosphate synthase
	guanine deaminase
	glycine dehydrogenase
aspartate family amino acid metabolic process	L-serine dehydratase
	homocysteine synthase
	L-asparaginase
	---NA---
	threonine dehydratase
	benzoate 4-monooxygenase cytochrome p450
	dimethylglycine oxidase
	argininosuccinate synthase
	threonine ammonia-lyase precursor
	4-aminobutyrate aminotransferase
	aldehyde dehydrogenase
	3-hydroxyacyl-NAD binding
	D-amino-acid oxidase
	s-adenosylmethionine synthetase
	phosphoadenosine phosphosulfate reductase
	phosphoadenosine phosphosulfate reductase
	aminobenzoyl-glutamate utilization protein b
	5-methyltetrahydropteroyltriglutamate-homocysteine methyltransferase
cellular amino acid catabolic process	dimethylglycine oxidase
	NAD-specific glutamate dehydrogenase
	succinate-semialdehyde dehydrogenase
	4-aminobutyrate aminotransferase
	NAD-specific glutamate dehydrogenase
	proline oxidase
	aldehyde dehydrogenase
	methylmalonate-semialdehyde dehydrogenase
	3-hydroxyacyl-NAD binding
	carbamoyl-phosphate synthase
	succinate-semialdehyde dehydrogenase
	glycine dehydrogenase
gamma-aminobutyric acid catabolic process	succinate-semialdehyde dehydrogenase
	4-aminobutyrate aminotransferase
	succinate-semialdehyde dehydrogenase

### Modulation of fungal GO categories during infection of WT tomato lines

Upregulation of the GABA (Gamma-Aminobutyric Acid) metabolic process in *C. gloeosporioides* while infecting the WT suggested its contribution as a carbon and nitrogen source to spore/sporulation metabolism, as described for other fungi under similar pH conditions [59, 60]. GABA synthesis has been associated with acidic pH in different plant and fungal systems [59], either in response to cytosolic acidification—probably as a pH-regulatory mechanism, or during growth under acidic conditions [59]. Enhanced GABA synthesis might be one of the mechanisms employed by the fungus to utilize carbon and nitrogen sources under the acidic pH of the WT tomato [61], and it is consequently not expressed in the neutral RNAi*–SlPH* line.

#### Carbohydrate active enzymes (CAZymes)

The diverse complex carbohydrates that contribute to *C. gloeosporioides* virulence are controlled by a panel of enzymes involved in their assembly (glycosyltransferases) and breakdown (glycoside hydrolases, polysaccharide lyases, carbohydrate esterases), collectively designated CAZymes [62]. In plant pathogens, CAZymes promote synthesis, degradation, and modification of carbohydrates, playing an important role in the breakdown of plant cell walls and in host–pathogen interactions [63]. *C. gloeosporioides* uses different sets of enzymes to attack the tomatoes with the different pHs by degrading complex carbohydrates of the host to simple monomers that can be utilized as nutrients [64, 65].

**Fig. 5:**
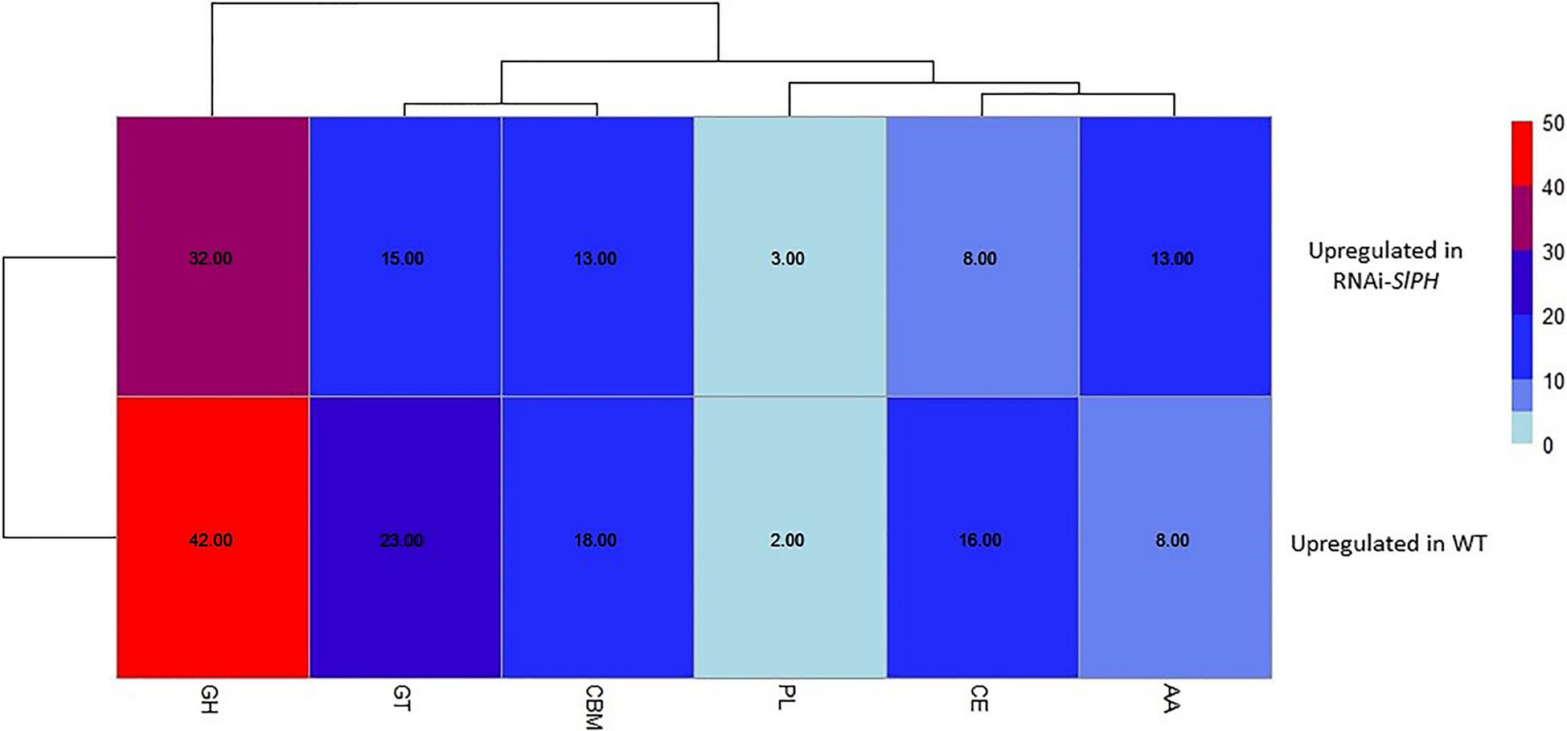
Heat map of numbers of carbohydrate enzyme (CAZyme) repertoires which were found to be differentially expressed. GH – glycoside hydrolase; GT – glycosyl transferase; PL – polysaccharide lyase; CE – carbohydrate esterase; AA – auxiliary activities; CBM – carbohydrate-binding modules. Upregulated genes represent genes with higher expression levels in RNAi–*SlPH* compared to the WT *line*. Downregulated genes represent lower expression levels in RNAi–*SlPH* vs. the WT *line*

Using the CAZyme analysis toolkit [66], we identified 80 upregulated CAZymes during infection of line RNAi–*SlPH* and 107 upregulated CAZymes during infection of the WT line (Fig. 5). This indicates that similar numbers of genes encoding CAZyme activities were regulated in both tomato lines, suggesting that the number of differentially expressed CAZymes is not the limiting factor in increased colonization of the RNAi–*SlPH* compared to the WT line by *C. gloeosporioides.*

### Validation of host and fungal gene expression

**Fig. 6:**
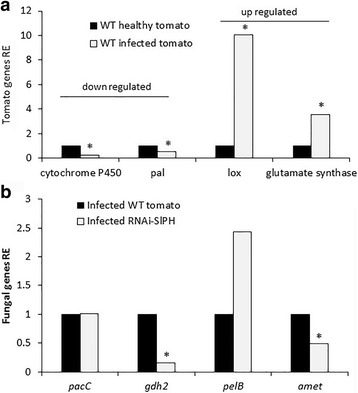
Validation of RNA-Seq results by qRT-PCR. Eight differentially expressed genes (four from the host and four from the fungus) were examined by qRT-PCR at the same time points analyzed by the RNA-Seq. **a** Tomato genes of healthy vs. *C. gloeosporioides*-colonized WT tissue. Cytochrome P_450_ and phenylalanine ammonia lyase (*pal*) were downregulated while lipoxygenase (*lox*) and glutamate synthase were upregulated. Values were normalized to the values obtained from healthy tissue. **b** Fungal genes from the *C. gloeosporioides* colonizing WT vs. RNAi–*SlPH* tissue. *pacC* showed no differential effect, NAD^+^-specific glutamate dehydrogenase (*gdh2*) and ammonia transporter (*amet*) were downregulated during infection of the RNAi–*SlPH* line, and pectate lyase B (*pelB*) was upregulated during infection of the RNAi–*SlPH* line. Values were normalized to those obtained with the infected WT line. Primer list is provided in Additional file 1

To validate the differential expression of specific genes identified by the RNA-Seq analysis, quantitative (q) RT-PCR analyses were performed for key genes of interest. Analysis of the WT tomato lines showed the expected increase in expression of the LOX and glutamate synthase genes that modulated the relative resistance of the WT line compared to the RNAi–*SlPH* line. In contrast, the WT line showed downregulated expression of genes encoding cytochrome P_450_ and PAL related to downregulation of the phenylpropanoid pathway in the WT, which also explains the reduced colonization of *C. gloeosporioides* on the WT line (Fig. 6a).

An interesting outcome of the comparison of *C. gloeosporioides* colonization on the WT vs. RNAi–*SlPH* line is the similar expression pattern of *pacC* in the fungus on both lines, indicating that the lower pH of the WT line increases when it is inoculated, as detected by the accumulation of ammonia (Fig. 1), to a value similar to that of the RNAi–*SlPH* line, leading to similar expression of *pacC* during colonization. However, the enhanced susceptibility of RNAi–*SlPH* was accompanied by upregulation of *pelB* and in contrast, downregulation of glutamate degradation, as reflected by the downregulation of *gdh2* and the ammonia transporter *amet* (Fig. 6b), further explaining the reduced ammonia accumulation in the RNAi–*SlPH* line.

## Conclusions

*SlPH* has been found to play a significant role in tomato fruit acidity, with its modulation of tomato pH levels [1]. The transgenic RNAi–*SlPH* tomato showed reduced organic acid accumulation and a higher pH level than the WT tomato, and it was more susceptible to *C. gloeosporioides* attack. Our aim was to analyze the effect of the fruit’s initial pH on the host and pathogen gene profiles during fungal colonization. Gene-expression profiling revealed that fruit pH affects the host’s defense strategy as well as the fungus’ pathogenesis strategy.

Each host followed a different strategy in response to fungal attack. One of the key processes upregulated in the infected WT vs. RNAi–*SlPH* tomatoes was the 13-LOX pathway, resulting in JA biosynthesis, a known host defense response. Interestingly, the glutamate biosynthesis IV pathway was upregulated, while the glutamate degradation II pathway was downregulated. We suggest that the pathogen induced production of glutamate by the plant as a preferred nitrogen source for the fungus to accumulate ammonia, and to increase the environmental pH for better expression of the pathogenicity factors *pelB* and *pacC*. This may indicate that the host–pathogen interaction affects the host metabolism according to acid content and pH.

In contrast, the RNAi–*SlPH* tomato showed different modulated pathways. There were significant changes in sugar metabolism, in particular downregulation of glyoxylate degradation, which might have led to reduced organic acid accumulation in the RNAi–*SlPH* line and might have caused cellular and developmental problems, affecting host susceptibility. This suggests that modulation of the glyoxylate cycle contributes to this line’s susceptibility to *C. gloeosporioides*. Furthermore, the phenylpropanoid pathway showed differential modulation in the two lines: upregulation during infection of the RNAi–*SlPH* line and downregulation during infection of the WT line. This pathway may eventually lead to cell death, improving fungal colonization ability in the necrotrophic stage. This may contribute to the RNAi–*SlPH* line’s susceptibility and the WT’s resistance to *C. gloeosporioides* pathogenicity. Thus, the present work indicates that environmental pH has a significant effect on the “balance” of activated pathways and mechanism (s) leading to the final host response, and pathogen colonization outcome.

Gene-expression analysis of the fungal transcriptome revealed that the fungus also responds differently to each tomato line. Upregulation of the nitrogen metabolic process, involving nitrate/nitrite reductase, was a key induced factor in the pathogen colonizing the RNAi–*SlPH* line. These genes contribute to *Colletotrichum* pathogenicity [13]. On the other hand, the nitrogen transport process and GABA metabolism were upregulated in *C. gloeosporioides* colonizing the WT line. In this case, several genes involved in ammonia transport contributed to *C. gloeosporioides* pathogenicity (*gdh2, amet, mepB*) [9, 57].

In summary, enhanced colonization of tissue via pH modulation does not solely affect fungal pathogenicity. This is the first report of pH modulation—effected either locally by the fungus or genetically by affecting the expression of a single gene—determining the strategic response of the host to the challenging *Colletotrichum* colonization. On the pH-neutral RNAi–*SlPH* tomato, the major pathogenicity factors are probably modulated directly by PACC and include *pelB* expression, which may increase virulence compared to the WT acidic line where the fungus has to increase the pH using several ammonia transporters to activate pathogenicity factors in the postharvest pathogen.

## Methods

### Fungal isolates, plant cultivars and fruit inoculation

Single-spore cultures of *Colletotrichum gloeosporioides* (Penz.) isolate Cg-14 were obtained from a decayed avocado fruit [67]. The isolate was thoroughly analyzed in tomato infections [25]. Cultures were grown at 27 °C in the dark, and maintained on M3S plates. Conidia were harvested with 10 ml sterile distilled water supplemented with 0.01% (*v*/v) Triton X-100. Cells were visualized with a model BX60F-3 microscope (Olympus America Inc., Melville, NY, USA) and counted with a hemocytometer (Brand, GMBH, Wertheim, Germany).

Tomato (*Solanum lycopersicum* var. MP-1) plants were grown in the greenhouse under standard conditions. Transgenic *SlPH* tomatoes, impaired in the PH ortholog and obtained as described by Cohen et al. [1], and the WT strain were compared.

Tomato fruit was inoculated by placing 5 μl of a conidial suspension containing 10^6^ spore ml^−1^ on each of four 2-mm deep and 1-mm diameter wounds spaced evenly in a circle around the upper part of the stem end of 7–10 fruits. Following inoculation, the fruits were incubated for 72 h (unless otherwise specified) at 25 °C in covered plastic containers containing wet paper towels. Samples of the colonized tissue of the infected fruit were collected and frozen with liquid nitrogen for subsequent RNA analysis. Control tissue was obtained from healthy fruits of the RNAi–*SlPH* and WT lines.

To determine pH and acidity content in the fruit, juice from four to five tomatoes (20 g tissue from each tomato in four replicates) was extracted using a mortar and pestle and filtered through a gauze pad. Fruit pH was determined in the juice. Subsequently, acid content of the fruit juice was determined using a Metrohm titrator (678 EP/KF processor, Switzerland).

### Analysis and quantification of total ammonia production and pH changes effected by *C. gloeosporioides*

Tomato tissue pH was measured in the homogenized solution with a Hamilton double-pore slim electrode connected to a Thermo Orion Model 720A Plus pH meter. Ammonia was detected calorimetrically with an ammonium test kit (Merck, Darmstadt Germany); its concentration was determined in 5-ml samples of 10-fold-diluted supernatant of the homogenized fruit tissue, according to the manufacturer’s instructions. Ammonia concentration was analyzed in the healthy and colonized tissue, using healthy tissue extract as a control for the amount of ammonia present in the colonized tissue of the fruit. Concentrations were reported in millimolar ammonia per gram fresh weight (FW).

### RNA extraction

Total RNA was extracted from tomato fruit according to Yang et al. [68], with minor modifications: aliquots were taken from pooled samples of four to five inoculation areas on each tomato. The samples were ground to a fine powder in liquid nitrogen and transferred into 50-ml centrifuge tubes with 10 ml cetyltrimethylammonium bromide-RNA extraction buffer as described by [68]. The mixture was shaken for 3 min and then incubated at 65 °C for 15 min. The centrifuged sample was subjected to a second extraction under similar conditions. Following centrifugation, LiCl was added to a final concentration of 2.5 M and RNA was allowed to precipitate overnight at 4 °C. The precipitated RNA was pelleted at 4 °C for 30 min at 10,000×*g*, washed with 70% ethanol, and resuspended at 65 °C for 3 min in SSTE buffer comprised of 10 mM Tris pH 8, 1 M NaCl, 1 mM EDTA pH 8, and 0.5% (*w*/*v*) SDS and the samples were further treated as described before [50, 68]. Finally the RNA was further treated with Turbo DNAse (Ambion, Austin, TX, USA) and kept at −80 °C until use.

### Preparation of libraries

A 500-ng aliquot of total RNA from each of eight samples was processed with the Truseq Stranded Total RNA with Ribo Zero Plant (RS-122-2401) (Illumina, San Diego, CA, USA). Libraries were evaluated with Qubit and TapeStation software (Agilent Technologies, Palo Alto, CA, USA), and sequence libraries were constructed with barcodes to enable sample multiplexing. The results showed 13.2–16.9 million single-end 50-bp reads. These were sequenced on one lane of an Illumina HiSeq 2500 V3 instrument at the Bioinformatics Unit of the Weizmann Institute of Science, Rehovot, Israel.

### Bioinformatics analysis of RNA-Seq data

Eight libraries of single-end, 50-nucleotide-long total RNA-Seq reads were generated using Illumina HiSeq 2500. The libraries contained the following sequences: (i) duplicates of healthy WT tomato tissue, line 6421, with 15,127,651 and 13,235,200 reads, respectively; (ii) duplicates of healthy transgenic tomato tissue, line 6422, with 16,905,007 and 14,389,826 reads, respectively; (iii) duplicates of *C. gloeosporioides*-infected WT tomato tissue, line 6421, with 15,292,125 and 15,272,104 reads, respectively; (iv) duplicates of *C. gloeosporioides*-infected transgenic tomato tissue, line 6422, with 14,749,785 and 16,162,329 reads, respectively. The datasets are available at the NCBI Sequence Read Archive (SRA) under accession no. SRP078571.

Genome assemblies of tomato (*S. lycopersicum*, build 2.50 [23]) and *C. gloeosporioides* isolate Cg-14 (GEO accession no. GSE41844 [24]) were used as reference sequences. Bowtie2 software was used to align the RNA-Seq outputs against the transcriptome of *C. gloeosporioides* [26]. The libraries were aligned against the *C. gloeosporioides* genome and tomato genome (downloaded from NCBI accession no. PRJNA176412).

RSEM software was used for transcript quantification and the edgeR package for calculation of the differential gene expression for of the RNA-Seq data [27, 69]. The annotations of *C. gloeosporioides* were taken from GEO accession no. GSE41844 [24]. GO-enrichment was analyzed by Fisher’s Exact Test with multiple testing correction of FDR [70]. We compared the infected WT line with the infected RNAi–*SlPH* line, and therefore ‘upregulated in RNAi–*SlPH*’ means higher expression of the corresponding transcripts in the RNAi–*SlPH* line as compared to the WT line. Heat map and clustering of the genes were visualized by using the R software ggplots2 package [71].

We used a FDR threshold of <0.05 and logFC greater than 1 or smaller than −1 for analysis of *C. gloeosporioides* genes, and a FDR threshold of <1e-3 and logFC greater than 2 and smaller than −2 for tomato genes.

### Gene-expression analysis by qRT-PCR

Real-time qRT-PCR was performed with the StepOnePlus System (Applied Biosystems, Grand Island, NY, USA). PCR amplification was performed with 3.4 μl of cDNA template in 10 μl reaction mixture containing 6.6 μl mix from the SYBR Green Amplification Kit (ABgene, Surrey, UK) and 300 nM primers. Additional file 1 lists the forward and reverse primers for each of the indicated genes. The PCR was carried out as follows: 10 min at 94 °C, and 40 cycles of 94 °C for 10 s, 60 °C for 15 s, and 72 °C for 20 s. The samples were subjected to melting-curve analysis: efficiencies were close to 100% for all primer pairs, and all products showed the expected sizes of 70 to 100 bp. All of the samples were normalized to endogenous control gene-expression levels, and the values were expressed as the change (increasing or decreasing) in level relative to a calibrator sample. Results were analyzed with StepOnePlus software v.2.2.2. Relative quantification was performed by the ∆∆C_T_ method. The ∆C_T_ value was determined by subtracting the C_T_ results for the target gene from those for the endogenous control gene—*18S* for *C. gloeosporioides* and *TIP41* for tomato analysis—and normalizing against the calibration sample to generate the ∆∆C_T_ values. Each experiment was performed in triplicate, and three different biological experiments were conducted. One representative set of results is presented as mean values of 2^-∆∆CT^ ± SE for each treatment.

## Additional file

Additional file 1: Table S1. Primers for validations. (DOCX 12 kb)

## Abbreviations

13-HPL pathway: 13-hydroperoxide lyase pathway
CAZymes: Carbohydrate active enzymes
GABA: Gamma-Aminobutyric Acid
GO: Gene ontology
GS–GOGAT: GLutamine synthetase–glutamine-oxoglutarate aminotransferase
JA: Jasmonic acid
LOX: Lipoxygenase
PAL: Phenylalanine ammonia-lyase
pg1: Polygalacturonase
SA: Salicylic acid
SRA: Sequence read archive

## References

[1] Cohen S, Itkin M, Yeselson Y, Tzuri G, Portnoy V, Harel-Baja R, Lev S, Sa’ar U, Davidovitz-Rikanati R, Baranes N. The PH gene determines fruit acidity and contributes to the evolution of sweet melons. Nat Commun. 2014;5 10.1038/ncomms5026 24898284

[2] Davidzon M, Alkan N, Kobiler I, Prusky D (2010). Acidification by gluconic acid of mango fruit tissue during colonization via stem end infection by *Phomopsis mangiferae*. Postharvest Biol Technol.

[3] Eshel D, Lichter A, Dinoor A, Prusky D (2002). Characterization of *Alternaria alternata* glucanase genes expressed during infection of resistant and susceptible persimmon fruits. Mol Plant Pathol.

[4] Eshel D, Miyara I, Ailing T, Dinoor A, Prusky D (2002). pH regulates endoglucanase expression and virulence of *Alternaria alternata* persimmon fruit. Mol Plant-Microbe Interactions.

[5] Manteau S, Abouna S, Lambert B, Legendre L (2003). Differential regulation by ambient pH of putative virulence factor secretion by the phytopathogenic fungus *Botrytis cinerea*. FEMS Microbiol Ecol.

[6] Prusky D, McEvoy JL, Leverentz B, Conway WS (2001). Local modulation of host pH by *Colletotrichum* species as a mechanism to increase virulence. Mol Plant-Microbe Interactions.

[7] Prusky D, McEvoy JL, Saftner R, Conway WS, Jones R (2004). Relationship between host acidification and virulence of *Penicillium* spp. on apple and citrus fruit. Phytopathology.

[8] Miyara I, Shafran H, Davidzon M, Sherman A, Prusky D (2010). pH regulation of ammonia secretion by *Colletotrichum gloeosporioides* and its effect on appressorium formation and pathogenicity. Mol Plant-Microbe Interact.

[9] Miyara I, Shnaiderman C, Meng X, Vargas WA, Diaz-Minguez JM, Sherman A, Thon M, Prusky D (2012). Role of nitrogen-metabolism genes expressed during pathogenicity of the alkalinizing *Colletotrichum gloeosporioides* and their differential expression in acidifying pathogens. Mol Plant-Microbe Interact.

[10] Rollins JA, Dickman MB (2001). PH signaling in *Sclerotinia sclerotiorum*: identification of a pacC/RIM1 homolog. Appl Environ Microbiol.

[11] Bateman D, Beer S. Simultaneous production and synergistic action of oxalic acid and polygalacturonase during pathogenesis by Sclerotium rolfsii. Phytopathology. 1965;55(11)204–11. 14274523

[12] Jennings DH, Boddy L, Machant R, Read DJ (1989). Some perspectives on nitrogen and phosphorus metabolism in fungi. Nitrogen, phosphorus and sulphur utilization by fungi.

[13] Alkan N, Fluhr R, Sherman A, Prusky D (2008). Role of ammonia secretion and pH modulation on pathogenicity of *Colletotrichum coccodes* on tomato fruit. Mol Plant-Microbe Interact.

[14] Dieguez-Uribeondo J, Forster H, Adaskaveg JE (2008). Visualization of localized pathogen-induced pH modulation in almond tissues infected by *Colletotrichum acutatum* using confocal scanning laser microscopy. Phytopathology.

[15] O'Connell RJ, Thon MR, Hacquard S, Amyotte SG, Kleemann J, Torres MF, Damm U, Buiate EA, Epstein L, Alkan N (2012). Lifestyle transitions in plant pathogenic *Colletotrichum* fungi deciphered by genome and transcriptome analyses. Nat Genet.

[16] Alkan N, Davydov O, Sagi M, Fluhr R, Prusky D (2009). Ammonium secretion by *Colletotrichum coccodes* activates host NADPH oxidase activity enhancing host cell death and fungal virulence in tomato fruits. Mol Plant-Microbe Interact.

[17] Kramer-Haimovich H, Servi E, Katan T, Rollins J, Okon Y, Prusky D (2006). Effect of ammonia production by *Colletotrichum gloeosporioides* on pelB activation, pectate lyase secretion, and fruit pathogenicity. Appl Environ Microbiol.

[18] Prusky D, Alkan N, Mengiste T, Fluhr R. Quiescent and Necrotrophic lifestyle choice during postharvest disease development. Annu Rev Phytopathol. 2013; 10.1146/annurev-phyto-082712-102349 23682917

[19] Barad S, Sela N, Kumar D, Kumar-Dubey A, Glam-Matana N, Sherman A, Prusky D (2016). Fungal and host transcriptome analysis of pH-regulated genes during colonization of apple fruits by *Penicillium expansum*. BMC Genomics.

[20] Prusky D, Gold S, Keen NT (1989). Purification and characterization of an endopolygalacturonase produced by *Colletotrichum gloeosporioides*. Physioll Molr Plant Path.

[21] Yakoby N, Beno-Moualem D, Keen NT, Dinoor A, Pines O, Prusky D (2001). *Colletotrichum gloeosporioides* pelB is an important virulence factor in avocado fruit-fungus interaction. Mol Plant-Microbe Interact.

[22] Yakoby N, Kobiler I, Dinoor A, Prusky D (2000). pH regulation of pectate lyase secretion modulates the attack of *Colletotrichum gloeosporioides* on avocado fruits. Appl Environ Microbiol.

[23] Sato S, Tabata S, Hirakawa H, Asamizu E, Shirasawa K, Isobe S, Kaneko T, Nakamura Y, Shibata D, Aoki K (2012). The tomato genome sequence provides insights into fleshy fruit evolution. Nature.

[24] Alkan N, Meng X, Friedlander G, Reuveni E, Sukno S, Sherman A, Thon M, Fluhr R, Prusky D (2013). Global aspects of *pacC* regulation of pathogenicity genes in *Colletotrichum gloeosporioides* as revealed by transcriptome analysis. Mol Plant-Microbe Interact.

[25] Alkan N, Friedlander G, Ment D, Prusky D, Fluhr R (2015). Simultaneous transcriptome analysis of Colletotrichum gloeosporioides and tomato fruit pathosystem reveals novel fungal pathogenicity and fruit defense strategies. The New Phytologist.

[26] Langmead B, Salzberg SL (2012). Fast gapped-read alignment with bowtie 2. Nat Methods.

[27] Robinson MD, McCarthy DJ, Smyth GK (2010). EdgeR: a bioconductor package for differential expression analysis of digital gene expression data. Bioinformatics.

[28] Joung J-G, Corbett AM, Fellman SM, Tieman DM, Klee HJ, Giovannoni JJ, Fei Z (2009). Plant MetGenMAP: an integrative analysis system for plant systems biology. Plant Physiol.

[29] Farmer EE, Ryan CA (1992). Octadecanoid precursors of jasmonic acid activate the synthesis of wound-inducible proteinase inhibitors. Plant Cell.

[30] Reymond P, Farmer EE (1998). Jasmonate and salicylate as global signals for defense gene expression. Curreent Opinion in Plant Biology.

[31] Kuhnl T, Koch U, Heller W, Wellmann E (1987). Chlorogenic acid biosynthesis: characterization of a light-induced microsomal 5-O-(4-coumaroyl)-D-quinate/shikimate 3′-hydroxylase from carrot (Daucus Carota L.) cell suspension cultures. Arch Biochem Biophys.

[32] Nicholson RL, Hammerschmidt R (1992). Phenolic compounds and their role in disease resistance. Annu Rev Phytopathol.

[33] Fry SC (1986). Cross-linking of matrix polymers in the growing cell walls of angiosperms. An Rev Plant Physiol.

[34] Schmidt A, Grimm R, Schmidt J, Scheel D, Strack D, Rosahl S (1999). Cloning and expression of a potato cDNA encoding hydroxycinnamoyl-CoA:tyramine N-(hydroxycinnamoyl) transferase. J Biol Chem.

[35] Cren M, Hirel B (1999). Glutamine synthetase in higher plants regulation of gene and protein expression from the organ to the cell. Plant Cell Physiol.

[36] Forde BG, Lea PJ (2007). Glutamate in plants: metabolism, regulation, and signalling. J Expl Bot.

[37] Lam H-M, Coschigano K, Oliveira I, Melo-Oliveira R, Coruzzi G (1996). The molecular-genetics of nitrogen assimilation into amino acids in higher plants. Annual Reviews of Plant Biology.

[38] Miflin BJ, Lea PJ (1976). The pathway of nitrogen assimilation in plants. Phytochemistry.

[39] Galili G, Tang G, Zhu X, Gakiere B (2001). Lysine catabolism: a stress and development super-regulated metabolic pathway. Curr Opin Plant Biol.

[40] Seifi HS, Van Bockhaven J, Angenon G, Höfte M (2013). Glutamate metabolism in plant disease and defense: friend or foe?. Mol Plant-Microbe Interact.

[41] Kang S, Kim HB, Lee H, Choi JY, Heu S, Oh CJ, Kwon SI, An CS (2006). Overexpression in Arabidopsis of a plasma membrane-targeting glutamate receptor from small radish increases glutamate-mediated Ca2+ influx and delays fungal infection. Molecules and Cells.

[42] Brauc S, De Vooght E, Claeys M, Geuns JM, Hofte M, Angenon G (2012). Overexpression of arginase in Arabidopsis Thaliana influences defence responses against Botrytis Cinerea. Plant Biology (Stuttgart, Germany).

[43] Tavernier V, Cadiou S, Pageau K, Laugé R, Reisdorf-Cren M, Langin T, Masclaux-Daubresse C (2007). The plant nitrogen mobilization promoted by Colletotrichum lindemuthianum in Phaseolus leaves depends on fungus pathogenicity. J Exp Bot.

[44] Hammond-Kosack KE, Parker JE (2003). Deciphering plant–pathogen communication: fresh perspectives for molecular resistance breeding. Curr Opin Biotechnol.

[45] Parniske M (2000). Intracellular accommodation of microbes by plants: a common developmental program for symbiosis and disease?. Curr Opin Plant Biol.

[46] Berg JM, Tymoczko JL, Stryer L: The glyoxylate cycle enables plants and bacteria to grow on acetate. Biochemistry. 5th edition. 2002. Section 17.4.

[47] Weber H, Chételat A, Reymond P, Farmer EE (2004). Selective and powerful stress gene expression in Arabidopsis in response to malondialdehyde. Plant J.

[48] Kotchoni SO, Kuhns C, Ditzer A, KIRCH HH, Bartels D (2006). Over-expression of different aldehyde dehydrogenase genes in Arabidopsis Thaliana confers tolerance to abiotic stress and protects plants against lipid peroxidation and oxidative stress. Plant, Cell and Environment.

[49] Allan WL, Clark SM, Hoover GJ, Shelp BJ (2009). Role of plant glyoxylate reductases during stress: a hypothesis. Biochem J.

[50] Bi F, Barad S, Ment D, Luria N, Dubey A, Casado V, Glam N, Mínguez JD, Espeso E, Fluhr R, et al. Carbon regulation of environmental pH by secreted small molecules that modulate pathogenicity in phytopathogenic fungi. Mol Plant Pathol. 2015; doi:10.1111/mpp.12355. 10.1111/mpp.12355 PMC6638356 26666972

[51] La Camera S, Gouzerh G, Dhondt S, Hoffmann L, Fritig B, Legrand M, Heitz T (2004). Metabolic reprogramming in plant innate immunity: the contributions of phenylpropanoid and oxylipin pathways. Immunol Rev.

[52] Dixon RA, Achnine L, Kota P, Liu CJ, Reddy M, Wang L (2002). The phenylpropanoid pathway and plant defence—a genomics perspective. Mol Plant Pathol.

[53] Verberne MC, Verpoorte R, Bol JF, Mercado-Blanco J, Linthorst HJ (2000). Overproduction of salicylic acid in plants by bacterial transgenes enhances pathogen resistance. Nat Biotechnol.

[54] Dempsey DMA, Shah J, Klessig DF (1999). Salicylic acid and disease resistance in plants. Crit Rev Plant Sci.

[55] Shirasu K, Nakajima H, Rajasekhar VK, Dixon RA, Lamb C (1997). Salicylic acid potentiates an agonist-dependent gain control that amplifies pathogen signals in the activation of defense mechanisms. Plant Cell.

[56] Divon HH, Ziv C, Davydov O, Yarden O, Fluhr R (2006). The global nitrogen regulator, FNR1, regulates fungal nutrition-genes and fitness during Fusarium oxysporum pathogenesis. Mol Plant Pathol.

[57] Shnaiderman C, Miyara I, Kobiler I, Sherman A, Prusky D (2013). Differential activation of ammonium transporters during the accumulation of ammonia by *Colletotrichum gloeosporioides* and its effect on appressoria formation and pathogenicity. Mol Plant-Microbe Interact.

[58] Miyara I, Shafran H, Kramer Haimovich H, Rollins J, Sherman A, Prusky D (2008). Multi-factor regulation of pectate lyase secretion by *Colletotrichum gloeosporioides* pathogenic on avocado fruits. Mol Plant Pathol.

[59] Kumar S, Punekar NS (1997). The metabolism of 4-aminobutyrate (GABA) in fungi. Mycol Res.

[60] Mead O, Thynne E, Winterberg B, Solomon PS (2013). Characterising the role of GABA and its metabolism in the wheat pathogen *Stagonospora nodorum*. PLoS One.

[61] Sanchez-Torres P, Gonzalez-Candelas L (2003). Isolation and characterization of genes differentially expressed during the interaction between apple fruit and *Penicillium expansum*. Mol Plant Pathol.

[62] Lombard V, Golaconda Ramulu H, Drula E, Coutinho PM, Henrissat B (2014). The carbohydrate-active enzymes database (CAZy) in 2013. Nucleic Acids Res.

[63] Zhao Z, Liu H, Wang C, Xu J-R (2013). Comparative analysis of fungal genomes reveals different plant cell wall degrading capacity in fungi. BMC Genomics.

[64] Marcet-Houben M, Ballester A-R, de la Fuente B, Harries E, Marcos JF, González-Candelas L, Gabaldón T (2012). Genome sequence of the necrotrophic fungus Penicillium digitatum, the main postharvest pathogen of citrus. BMC Genomics.

[65] Cantarel BL, Coutinho PM, Rancurel C, Bernard T, Lombard V, Henrissat B (2009). The carbohydrate-active EnZymes database (CAZy): an expert resource for glycogenomics. Nucleic Acids Res.

[66] Park BH, Karpinets TV, Syed MH, Leuze MR, Uberbacher EC (2010). CAZymes analysis toolkit (CAT): web service for searching and analyzing carbohydrate-active enzymes in a newly sequenced organism using CAZy database. Glycobiology.

[67] Wattad C, Dinoor A, Prusky D (1994). Purification of pectate lyase produced by Colletotrichum gloeosporioides and its inhibition by epicatechin: a possible factor involved in the resistance of unripe avocado fruits to anthracnose. Mol Plant-Microbe Interact.

[68] Yang G, Zhou R, Tang T, Shi S (2008). Simple and efficient isolation of high-quality total RNA from Hibiscus Tiliaceus, a mangrove associate and its relatives. Prep Biochem Biotechnol.

[69] Li B, Dewey CN (2011). RSEM: accurate transcript quantification from RNA-Seq data with or without a reference genome. BMC Bioinformatics.

[70] Benjamini Y, Hochberg Y (1995). Controlling the false discovery rate - a practical and powerful approach to multiple testing. J Royal Stat Soc.

[71] Team RDC (2009). R: a language and environment for statistical computing.

[72] Oliveros JC. Venny. An interactive tool for comparing lists with Venn’s diagrams. (2007–2015). http://bioinfogp.cnb.csic.es/tools/venny/index.html. Accessed 9 Mar 2015.

