# Supplementary material for: Differential gene expression in tomato fruit and Colletotrichum gloeosporioides during colonization of the RNAi–SlPH tomato line with reduced fruit acidity and higher pH
Source: BMC Genomics. 2017 Aug 4;18:579. doi: 10.1186/s12864-017-3961-6 (PMC5545021; doi:10.1186/s12864-017-3961-6)
Supplement: Additional file 1: Table S1. — Primers for validations. (DOCX 12 kb) [file 12864_2017_3961_MOESM1_ESM.docx]

Supplementary table 1 – primers for validations

|  | **Primer name** | **Forward** | **Reverse** |
| --- | --- | --- | --- |
| ***Solanum lycopersicum*** | *cytochrome p450* | GAAACTAAGGTGGTTGTGAATGCA | TCTCAGGTCGGAACTCATTCG |
|  | *pal* | TGCTAATGTGCTCGCGGTAT | CAGTGAACTCGGGCTTTCCA |
|  | *glutamate synthase* | CATGCTGGCAGAGGAAGTGA | TGTCTGAACGGCCAACCA |
|  | *lox* | AATCAGATGACGCGGTACAGAA | CTTGTCGCCATGTCCTTCTTC |
|  | TIP41 | ATGGAGTTTTTGAGTCTTCTGC | GCTGCGTTTCTGGCTTAGG |
|  |  |  |  |
| ***Colletotrichum gloeosporioides*** | *18S* | GTGAGGCCCTCAAAAGGTA | GGATCCCAGTGCGGACG |
|  | *pacC* | CTGTCTACGAAAACGCGAACC | AAGGGCTGTTGCGGAGATC |
|  | *gdh2* | AGAATCGGCGCAACTCTGTT | CGTGTATCCCAGCCTTAACGAA |
|  | *pelB* | TCGAAGGAGACTGGATACGCTACT | CCTTGGGCGCAGTGTTCT |
|  | *amet* | TGGCGTCAAAATCTCGAAGA | GCGGAAACTCAATGCTCTGAA |
